# Genetic Analysis of Loop Sequences in the Let-7 Gene Family Reveal a Relationship between Loop Evolution and Multiple IsomiRs

**DOI:** 10.1371/journal.pone.0113042

**Published:** 2014-11-14

**Authors:** Tingming Liang, Chen Yang, Ping Li, Chang Liu, Li Guo

**Affiliations:** 1 Jiangsu Key Laboratory for Molecular and Medical Biotechnology, College of Life Science, Nanjing Normal University, Nanjing, 210023, China; 2 Department of Epidemiology and Biostatistics, School of Public Health, Nanjing Medical University, Nanjing, 211166, China; Beckman Research Institute of the City of Hope, United States of America

## Abstract

While mature miRNAs have been widely studied, the terminal loop sequences are rarely examined despite regulating both primary and mature miRNA functions. Herein, we attempted to understand the evolutionary pattern of loop sequences by analyzing loops in the let-7 gene family. Compared to the stable miRNA length distributions seen in most metazoans, higher metazoan species exhibit a longer length distribution. Examination of these loop sequence length distributions, in addition to phylogenetic tree construction, implicated loop sequences as the main evolutionary drivers in miRNA genes. Moreover, loops from relevant clustered miRNA gene families showed varying length distributions and higher levels of nucleotide divergence, even between homologous pre-miRNA loops. Furthermore, we found that specific nucleotides were dominantly distributed in the 5′ and 3′ terminal loop ends, which may contribute to the relatively precise cleavage that leads to a stable isomiR expression profile. Overall, this study provides further insight into miRNA processing and maturation and further enriches our understanding of miRNA biogenesis.

## Introduction

MicroRNAs (miRNAs), a class of small non-coding RNA, are widely studied as crucial regulatory molecules able to modulate broad regulatory networks at the post-transcriptional levels [1], [2]. miRNA is generated from primary miRNA (pri-miRNA) and precursor miRNA (pre-miRNA) in animals [3], with the pre-miRNA presenting a stable stem-loop structure. Both of the two arm products, miR-#-5p and miR-#-3p, have been reported to form mature and functional miRNAs [4]–[8], with the loop sequences connecting miR-#-5p and miR-#-3p in the stem-loop structure typically degraded during miRNA biogenesis. These transitory intermediates are rarely examined due to an unseen direct role in the miRNA regulation process. Instead, most studies have focused on the functional and regulatory roles of miRNAs during miRNA-mRNA recognition as it relates to expression or translational repression. However, indeed, both pri-miRNA and pre-miRNA may contribute to the regulatory process [9]. Specifically, the loop nucleotides may tune and alter miRNA activity, controlling the processing precision during the miRNA maturation process [9].

Recently, the potential impact of hairpin loops has been examined, with short hairpin RNA (shRNA) loops possibly influencing effectivity [10]–[12] and alternative loop conformations in miRNAs potentially effecting expression [13], [14]. The loop sequence may also affect Dicer recognition and possibly specificity, thus affect miRNA cleavage during the maturation process [11], [15], [16]. As mentioned above, the loop sequence in pre-miRNAs has an important role in regulating the activities and specificities of related molecules that can facilitate Drosha and Dicer, with a mutation in the loop possibly affecting miRNA processing [17], [18]. Furthermore, miRNAs have been a key focal point because a miRNA locus can yield multiple sequences have diverse 5′ and/or 3′ ends and expression levels [19]–[25]. These miRNA variants, or physiological miRNA isoforms (isomiRs), may be mainly generated by imprecise Drosha and Dicer cleavage during pre-miRNA processing [22]. Additionally, the loop sequences may also impact pre-miRNA processing by affecting Drosha and/or Dicer directly or indirectly. Even though pre-miRNA loop sequences have been shown to directly or indirectly impact Drosha and Dicer during miRNA processing, few studies have performed an evolutionary analysis of the loop sequences, especially one focused on homology and clustered miRNAs across different animal species.

Herein, we attempt to perform an evolutionary analysis of the loop sequences in let-7 and locate related miRNAs across different animal species. The let-7 gene family has been widely detected in metazoans, with its associated miRNAs thought to have co-expanded with the HOX gene clusters [26]. A series of homologous miRNAs can be found in a miRNA gene family, including multicopy pre-miRNAs from specific animal species. Simultaneously, some members can be located in a cluster with other related miRNAs, with miRNAs within a close proximity on a chromosome being co-transcribed as a polycistronic transcript [27]–[30]. Therefore, a classical miRNA gene family and related miRNAs are typically selected to track and reveal the evolutionary patterns of the ever present yet ignored loop sequences. Thus, this study will examine the roles of loops during miRNA maturation and processing.

## Materials and Methods

MiRNA members in the let-7 gene family, to include homologous miRNAs and miRNAs from different animal species, were obtained from the miRBase database (http://www.mirbase.org/cgi-bin/mirna_summary.pl?fam=MIPF0000002). According to the location distributions, some were located within a gene cluster and all pre-miRNA and miRNA let-7 sequences and other related miRNAs were simultaneously collected.

pre-miRNA loop sequences were collected, including miR-#-5p and miR-#-3p sequences. If the miR-#-5p or miR-#-3p sequences were not annotated in the miRBase database, the complementary antisense miRNA consensus sequences was collected following alignment with the Clustal X 2.0 software [31]. Despite the sequence diversity of miRNAs among different animal species, miRNAs are phylogenetically conserved, particularly in the functional seed sequences (nucleotides 2–8). miRNAs can also vary in their length distributions, which can be attributed to being derived from various 3′ ends. Additionally, multiple isomiRs have been widely detected from miRNA loci, which indicates that flexible 5′ and 3′ ends mediate these loop sequences. The present study mainly focused on the core loop sequences. The 5′ and 3′ ends of loop sequences are cleavage sites of Dicer, with previous studies showing that imprecise cleavage by Drosha and Dicer can lead isomiRs generation via biased cleavage [24], [32], [33]. Based on this phenomenon, in addition to biased cleavage, the continuous 5, 3 and 1 nucleotides in the 5′ and 3′ ends of the loop sequences were specifically analyzed. To understand the effect of changes in the pre-miRNA loop sequences, the minimum free energy of relevant pre-miRNA sequences were predicted using the RNAfold webserver [34].

Phylogenetic trees were constructed using pre-miRNAs and loop sequences were constructed based on the Neighbor-Joining (NJ) method using the MEGA 6.06 software [35]. Evolutionary relationships among loop sequences within the hsa-let-7 gene family were reconstructed using SplitsTree 4.10 [36]. Sequence logos of relevant loop sequences across homologous miRNAs or clustered miRNAs were analyzed using the WebLogo program (http://weblogo.berkeley.edu/logo.cgi) [37]. An un-paired *t* test was used to estimate length distribution differences between the two groups and the chi-square (*χ^2^*) test was performed to estimate differences in loop nucleotide composition. Length distribution differences among multiple groups were estimated using ANOVA analysis (*P*<0.05), followed by a *q* test if a significant *P*-values was obtained. Adjusted *P*-values were obtained based on pairwise comparison. All the relevant statistical analysis was performed using the Stata software (Version 11.0).

## Results

### Overview of the distribution and sequence characteristics of let-7 loop sequences

**Figure 1:**
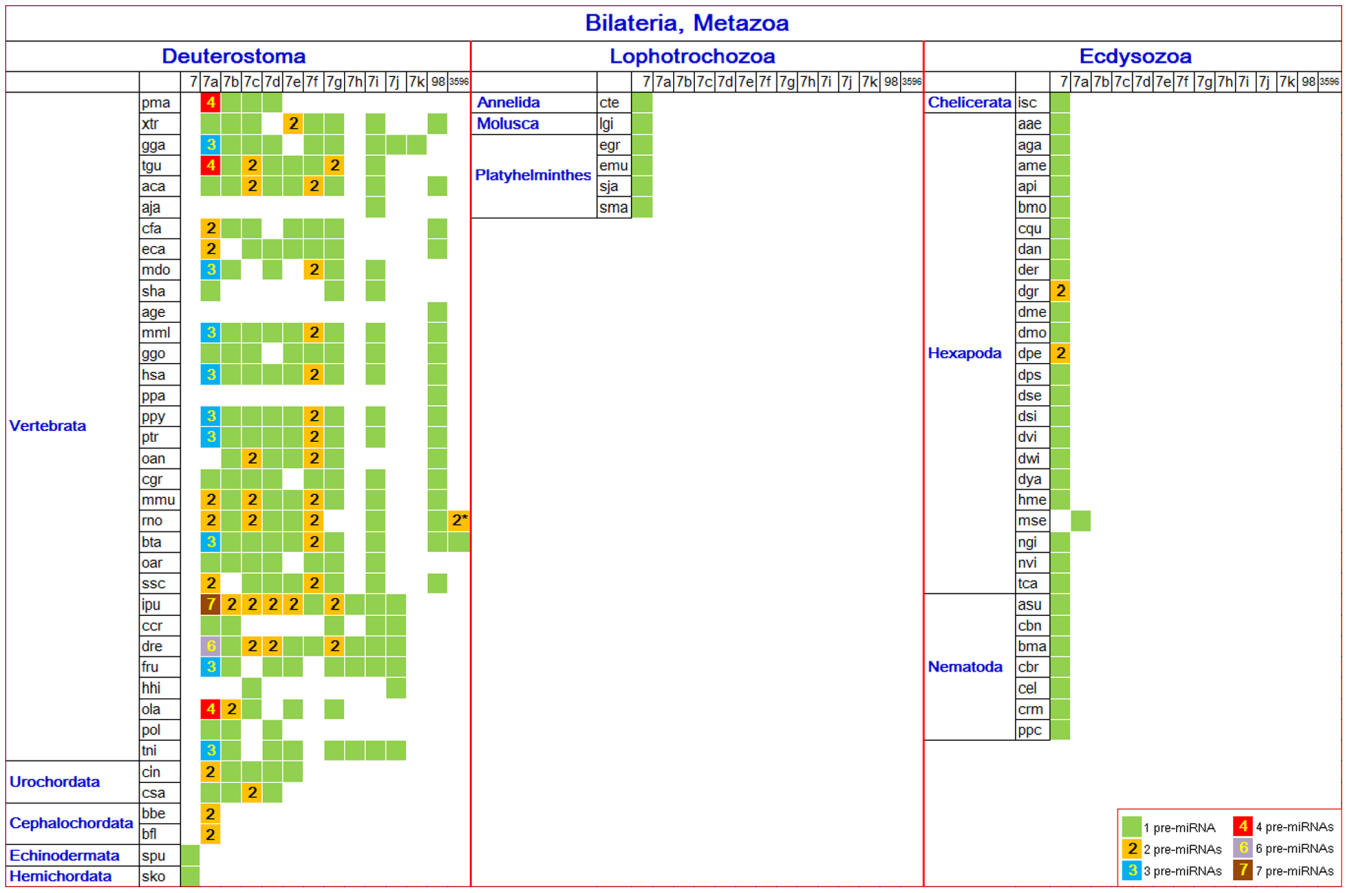
The distribution of the let-7 family among metazoan animals. The let-7 gene was named and homologous miRNAs were further found and named. These miRNA names were consistent with the current annotations in the miRBase database. “2*” indicates that there are two genes, rno-mir-3596b and rno-mir-3596d, and not two pre-miRNAs for the mature miRNA.

Let-7 gene family members have been found in 75 metazoan animal species (Figure 1). Multiple homologous miRNA genes, some being multicopy pre-miRNAs, were more prevalent in vertebrates and urochordates, while single miRNA genes were more common in other metazoan species (Figure 1). Among these miRNAs, let-7a showed higher pre-miRNA multicopy numbers (1–7) relative to other homologous miRNAs. Although these multicopy pre-miRNAs could generate the same let-7a-5p sequences, their loop sequences showed larger genetic distances (Figure S1 in File S1). Phylogenetic tree construction showed that the pre-miRNA multicopy loop sequences may be divided into two clusters based on loop and pre-miRNA sequences, with distribution differences noted (Figure S1 in File S1).

**Figure 2:**
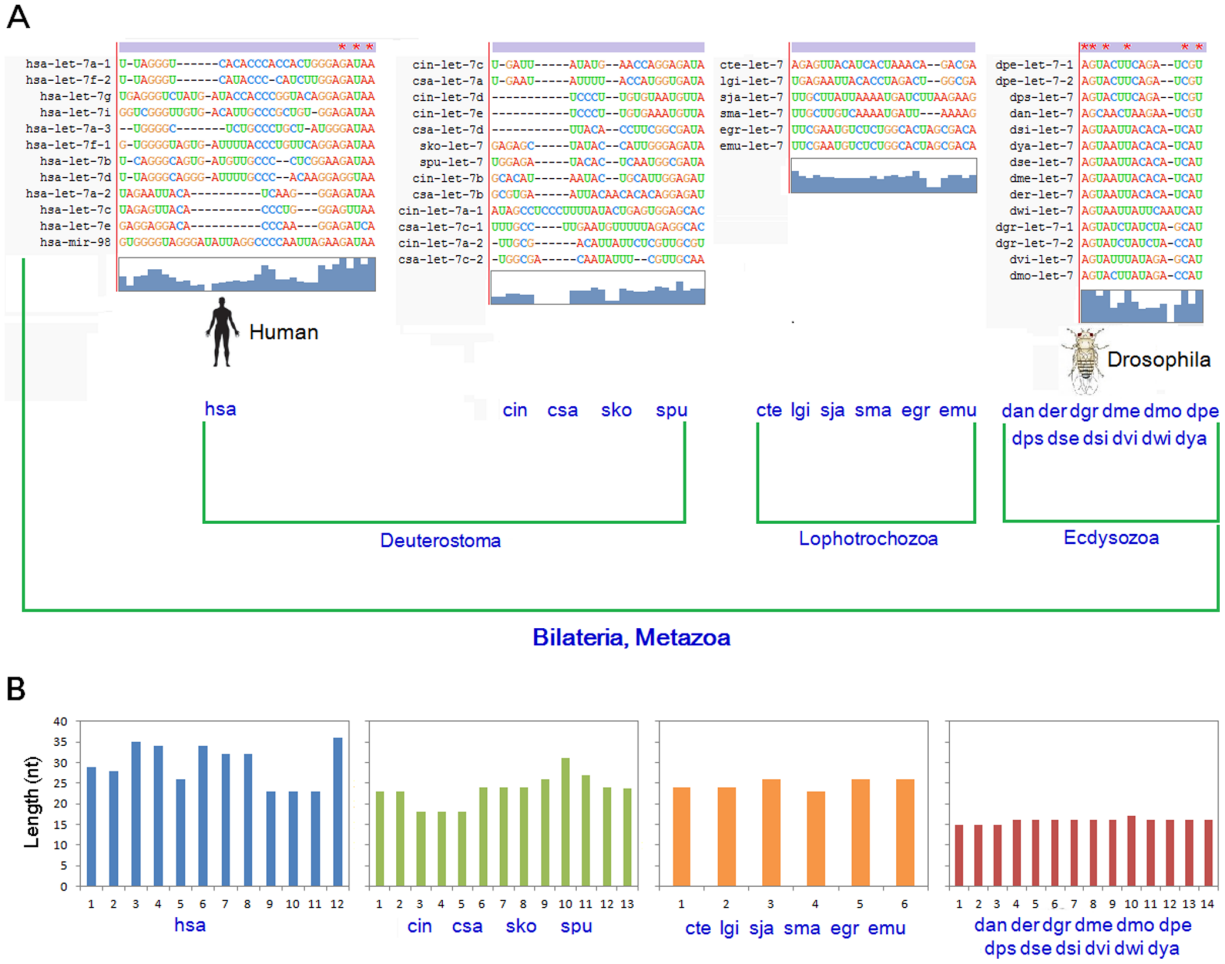
Sequence diversity and length distribution of the loop sequences among different animal species. (aae, *Aedes aegypti*; aca, *Anolis carolinensis*; aga, *Anopheles gambiae*; age, *Ateles geoffroyi*; aja, *Artibeus jamaicensis*; ame, *Apis mellifera*; api, *Acyrthosiphon pisum*; asu, *Ascaris suum*; bbe, *Branchiostoma belcheri*; bfl, *Branchiostoma floridae*; bma, *Brugia malayi*; bmo, *Bombyx mori*; bta, *Bos taurus*; cbn, *Caenorhabditis brenneri*; cbr, *Caenorhabditis briggsae*; ccr, *Cyprinus carpio*; cel, *Caenorhabditis elegans*; cfa, *Canis familiaris*; cgr, *Cricetulus griseus*; cin, *Ciona intestinalis*; cqu, *Culex quinquefasciatus*; crm, *Caenorhabditis remanei*; csa, *Ciona savignyi*; cte, *Capitella teleta*; dan, *Drosophila ananassae*; der, *Drosophila erecta*; dgr, *Drosophila grimshawi*; dme, *Drosophila melanogaster*; dmo, *Drosophila mojavensis*; dpe, *Drosophila persimilis*; dps, *Drosophila pseudoobscura*; dre, *Danio rerio*; dse, *Drosophila sechellia*; dsi, *Drosophila simulans*; dvi, *Drosophila virilis*; dwi, *Drosophila willistoni*; dya, *Drosophila yakuba*; eca, *Equus caballus*; egr, *Echinococcus granulosus*; emu, *Echinococcus multilocularis*; fru, *Fugu rubripes*; gga, *Gallus gallus*; ggo, *Gorilla gorilla*; hhi, *Hippoglossus hippoglossus*; hme, *Heliconius melpomene*; hsa, *Homo sapiens*; ipu, *Ictalurus punctatus*; isc, *Ixodes scapularis*; lgi, *Lottia gigantean*; mdo, *Monodelphis domestica*; mml, *Macaca mulatta*; mmu, *Mus musculus*; mse, *Manduca sexta*; ngi, *Nasonia giraulti*; nvi, *Nasonia vitripennis*; oan, *Ornithorhynchus anatinus*; oar, *Ovis aries*; ola, *Oryzias latipes*; pma, *Petromyzon marinus*; pol, *Paralichthys olivaceus*; ppa, *Pan paniscus*; ppc, *Pristionchus pacificus*; ppy, *Pongo pygmaeus*; ptr, *Pan troglodytes*; rno, *Rattus norvegicus*; sha, *Sarcophilus harrisii*; sja, *Schistosoma japonicum*; sko, *Saccoglossus kowalevskii*; sma, *Schistosoma mansoni*; spu, *Strongylocentrotus purpuratus*; ssc, *Sus scrofa*; tca, *Tribolium castaneum*; tgu, *Taeniopygia guttata*; tni, *Tetraodon nigroviridis*; xtr, *Xenopus tropicalis*).

Although loop sequences were believed to not be well-conserved relative to the miR-#-5p and miR-#-3psequences, loop sequences in Drosophila were found to be highly conserved (Figure 2A). Loop sequences among different species or from homologous miRNAs or multicopy pre-miRNAs showed higher levels of sequence divergence. Nucleotide insertions/deletions were common in the loop sequences, even between homologous miRNAs in the same species or between multicopy pre-miRNAs. Interestingly, varying length distributions between loop sequences from different animal species were noted (Figure 2B, *P*_adj_<0.05, except between Urochordata and Lophotrochozoa, Table S1 in File S1).

**Figure 3:**
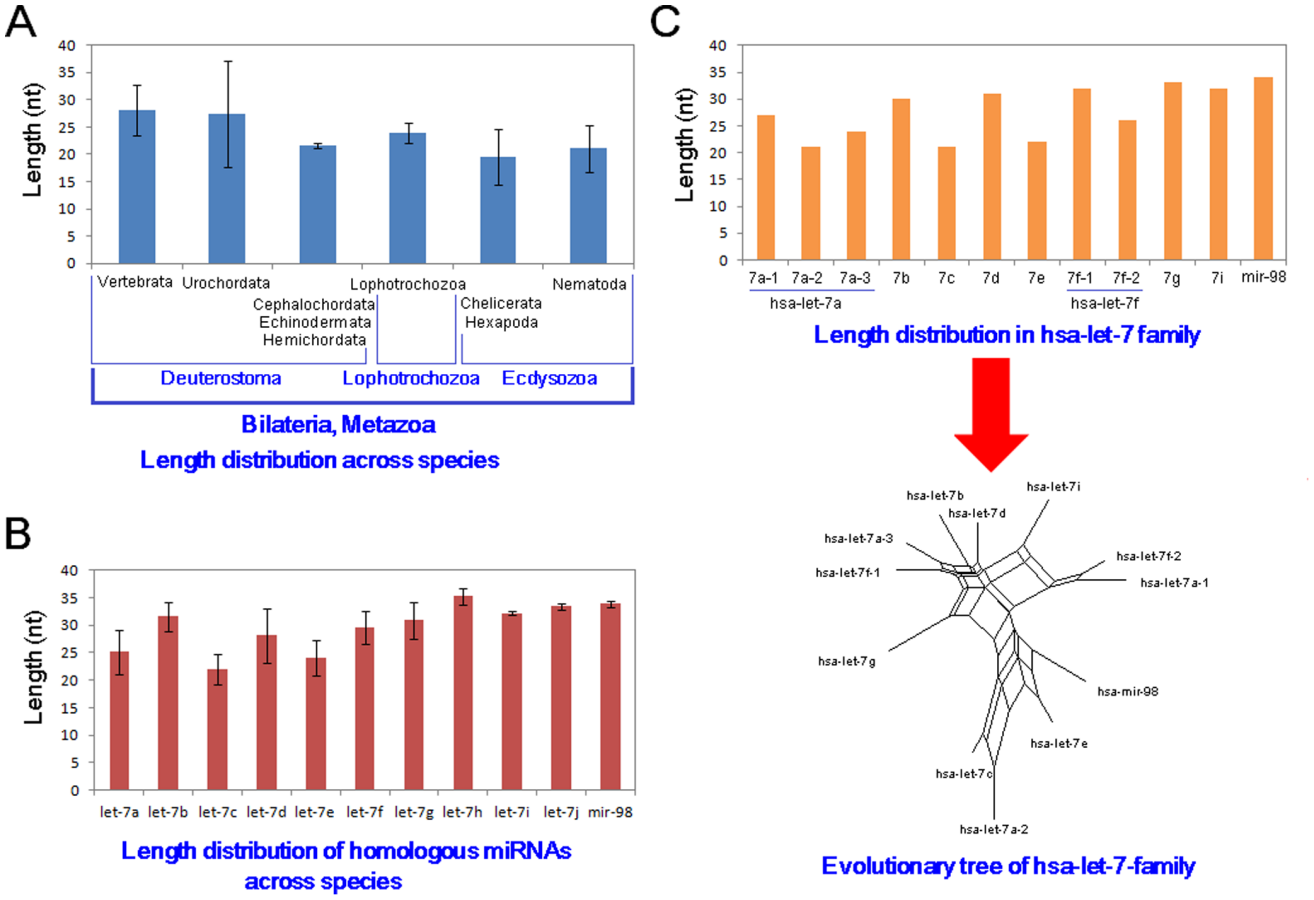
Length distributions of loops and their relevant evolutionary tree. (A) Length distribution of the let-7 loop among different animal species; (B) length distribution of loops in homologous miRNAs within the let-7 family across different animal species; and (C) length distribution of homologous hsa-let-7 loops and relevant evolutionary tree.

Based on the whole let-7 population, changes in length distribution across different species were found to be significant (*F* = 19.14, *P*<0.0001, Figure 3A). The longest average loop length was found in vertebrates (28.13±4.66 nts), followed by those in urochordates (27.36±9.78 nts), which were longer loop sequences than those found in other metazoans (Figure 3A). Each member in the let-7 gene family showed an inconsistent length distribution (Figure 3), despite being homologous miRNAs with higher sequence similarity. Specifically, let-7a (25.12±3.99 nts), let-7c (22.03±2.81 nts) and let-7e (24.17±3.21 nts) had shorter length distributions than other homologous miRNAs (from 28.12±4.87 nts to 35.25±1.50 nts) (*F* = 39.97, *P*<0.0001, Figure 3B). To further examine potential relationships between length distributions and evolutionary patterns, hsa-let-7 family loop sequences were analyzed. Loop sequences from multicopy pre-miRNAs showed larger divergence lengths, with these relevant loop sequences located in different evolutionary clusters (Figure 3C and Figure S1C in File S1). Larger genetic distances were noted in homologous miRNAs, with shorter loops from the let-7a-2, let-7c and let-7e genes located in a single cluster (Figure 3C and Figure S1C in File S1).

### Nucleotide characteristics in loop sequence

**Figure 4:**
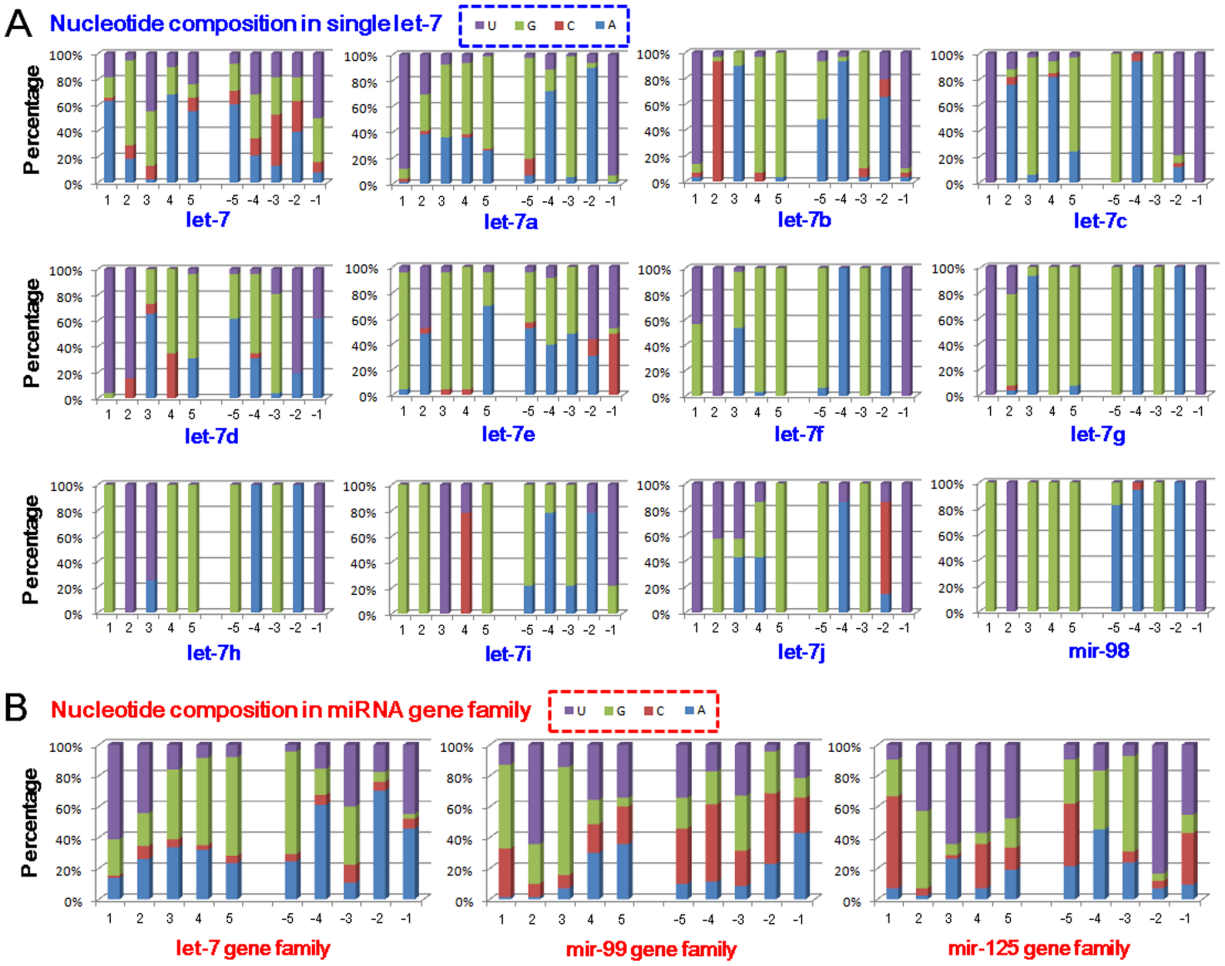
Nucleotide composition of the 5 continuous nucleotides at the 5′ and 3′ ends of the loop sequence. (A) Loop nucleotide composition in single let-7 genes (including let-7, let-7a, let-7b, etc.), with rare let-7 genes, such as let-7k and mir-3596, were not analyzed. (B) Loop nucleotide composition of relevant clustered miRNA gene families.

**Figure 5:**
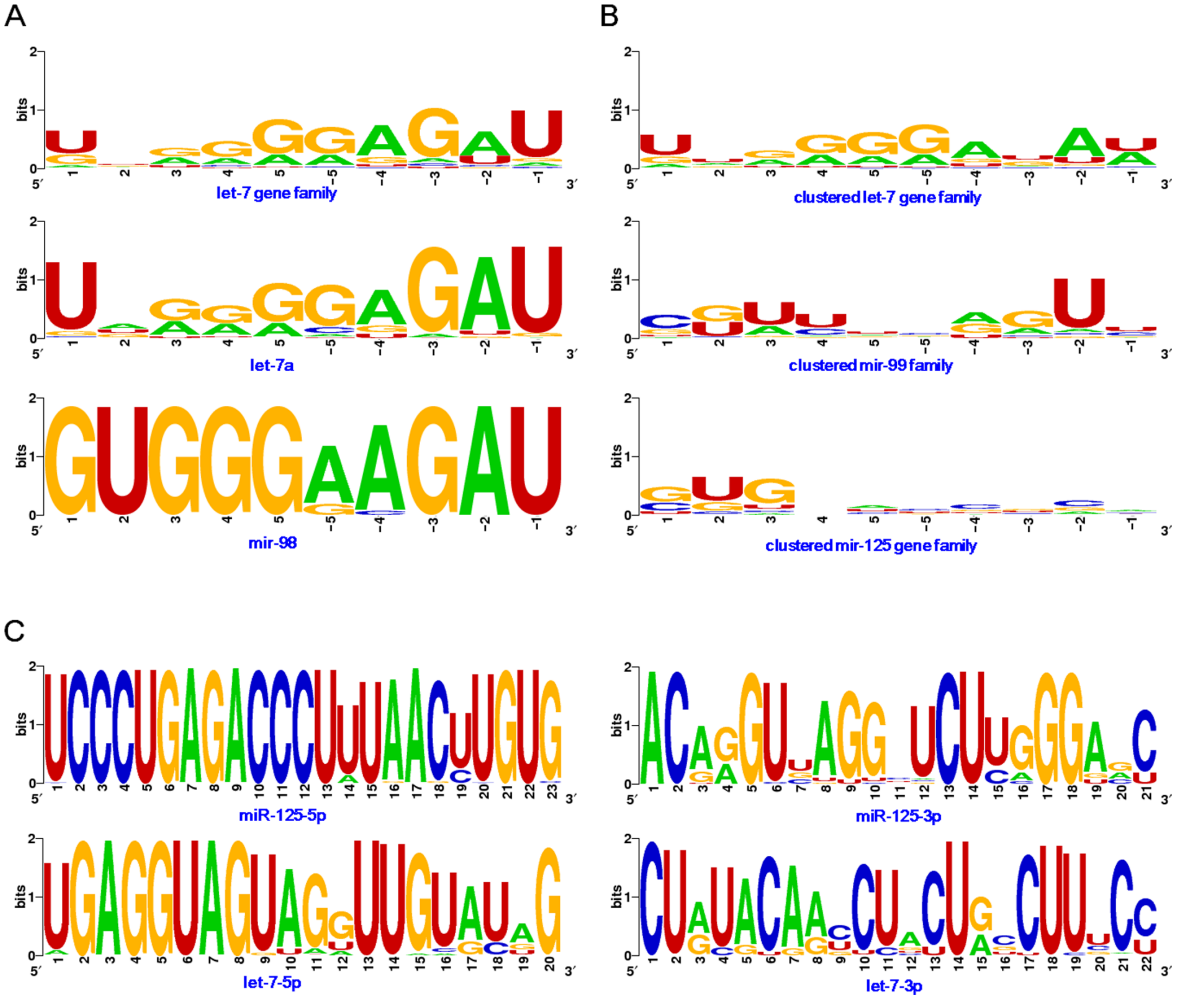
Sequence logo of loop sequences and miRNA sequences. (A) Sequence logo of all of the let-7 loop sequences, including the single let-7a and mir-98 loop sequences; (B) sequence logo of loop sequences from relevant clustered miRNA gene families; and (C) sequence logo of relevant mature miRNAs (including miR-#-5p and miR-#-3p).

According to the 10 nucleotides collected from the 5′ and 3′ ends of the loop sequences, guanine was the most prevalent (40.99%), followed by adenine (29.09%), while the cytosine presence was very low (5.32%) (Table S2 in File S1). Furthermore, if 3 continuous nucleotides were selected from either the 5′ or 3′ ends, no significant difference in nucleotide composition could be detected between the ends (*χ^2^* = 6.20, *P* = 0.102). However, significant differences could be found between the two ends if only one terminal nucleotide was analyzed (*χ^2^* = 63.90, *P*<0.0001). Uracil and guanine were the dominant nucleotides at the 5′ terminal ends (61.11% and 28.95%), while uracil was the most dominant nucleotide at the 3′ terminal ends (81.58%) (Figure 4, Table S2 in File S1 and Figure S2A in File S1). Guanine was present at an elevated rate, appearing at especially high frequencies in positions 4 and 5 at the 5′ ends and positions −3 and −5 at the 3′ ends (Table S2 in File S1), with a dominance also noted in the middle of the loop (Figure 5 and Figure S2A in File S1).

As a larger miRNA gene family, loop sequences showed larger nucleotide divergence than let-7-5p and let-7-3p (Figure 5A and Figure 5C). Let-7-5p acts as a canonical miRNA and is well conserved across animal species, particularly in the seed sequences. Among these homologous let-7 sequences, their loops showed varying levels of sequence similarity when examining single miRNA genes across animal species (Figure 5A and Figure S2A in File S1). While mature miRNAs, including miR-#-5p and miR-#-3p, are well conserved (especially for canonical mature miRNA) (Figure S2B in File S1), the loops commonly contain diverged nucleotides (Figure S2 in File S1) relative to the terminal regions that are relatively conserved.

### Loop sequences in related miRNAs clustered with let-7

Based on the close physical distance between let-7 and other miRNAs, relevant miRNAs clustered with let-7 were collected from different species. Similarly to the let-7 gene family, loops in related miRNA gene family were not as conserved (Figure 5B and Figure 5C). Interestingly, let-7 loops showed longer length distributions among their miRNA cluster (let-7 loops: 95% CI, 25.28–27.00 nts; clustered loops: 95% CI, 14.50–16.40 nts; *t* = 16.14, *P*<0.0001), with a similar relation seen among the human let-7 genes and its clustered miRNAs (Table S3 in File S1). Based on the 5 continuous nucleotides extending from the 5′ and 3′ ends, significant difference could be detected between the three physically distant relevant miRNA gene families (*χ^2^* = 301.41, *P*<0.0001). Most miRNA gene families showed divergence in the dominant nucleotides between 5′ and 3′ ends (Table S2 in File S1). Curiously, we found that the let-7 cluster showed inconsistent dominant nucleotides at the 3′ terminal across the let-7 gene family. Furthermore, diverse dominant nucleotides and nucleotide compositions could be detected between relevant alternative miRNA gene families (Table S2 in File S1).

## Discussion

miRNAs are generated from pre-miRNA through Dicer recognition and cleavage of the terminal loop [38], with the loop connecting to miR-#-5p and miR-#-3p, which may contribute to the hairpin activity [39]. As a class of small non-coding RNAs, the negative regulatory RNAs have been widely studied, but little focus has been placed on the loop sequences. Indeed, the loops, including the loop structure and sequence, may tune and alter miRNA activity [9], which may further affect miRNA expression [13], [14] by affecting Dicer recognition and cleavage during miRNA maturation [11], [15]. The potential roles of loop sequences during miRNA biogenesis have been studied, especially for occurrence of multiple isomiRs. These isomiRs are mainly derived from imprecise and alternative cleavage by Drosha and Dicer during miRNA processing and maturation [19]–[24]. These loop sequences may provide more information for miRNA biogenesis, especially based on the analysis of loop sequences across different animal species. In the present study, let-7 gene family loop sequences and other relevant miRNAs were analyzed and clustered to elucidate evolutionary and functional roles relating to miRNA biogenesis.

Multicopy miRNA genes may be located on different chromosomes and are mainly derived from historical duplication events [40]–[43]. Although the same mature miRNAs can be derived from these multicopy pre-miRNAs, the arms and loop sequences can be involved in larger divergence events, particularly the loops (Figure S1 in File S1) [7]. These loops may show diverse genetic distances relative to other homologous miRNA genes, and are always grouped in different clusters (Figure 3C and Figure S1 in File S1). Based on the phylogenetic relationships of pre-miRNAs and loops, similar distributions suggest that loop sequences dominate nucleotide divergence and evolutionary patterns in pre-miRNAs (Figure S1 in File S1). While both miR-#-5p and miR-#-3p sequences are conserved, loop sequences exhibit divergence through both varied nucleotides (including insertion/deletion) and lengths (Figure 2, Figure 5 and Figure S2 in File S1). In drosophila, the loop sequences and mature miRNAs are well conserved as well, with varied nucleotides and insertion/deletions found in the middle region of the loop (Figure 2A). The two terminal ends of the loops are relative conserved, which may contribute to stem-loop structure and cleavage by Dicer. These results suggest rapid evolution of the loops that further drives the evolution of miRNA genes.

Interestingly, we found changed length distributions of the loop sequences across different animal species and among different homologous miRNAs (Figure 2, Figure 3, Table S1 in File S1 and Table S3 in File S1). In higher metazoan species, the let-7 loops tend to be longer than those seen in lower species, with varying length distributions also seen different homologous miRNAs (Figure 3B and 3C). Clustered miRNAs tend to have similar length distribution, which implicates that the loop lengths may be affected by evolutionary relationships (Figure 3 and Figure S1 in File S1). Additionally, longer loop sequences may be an evolutionary trend in let-7 gene family, which may be of importance during miRNA biogenesis. Specifically, loop length may influence the stem-loop structure and stability (Table S4 in File S1), with longer loop sequences providing a possibility to dominate the evolution of miRNA genes across different animal species and homologous miRNAs within a specific species. These length variances further increase loop sequence diversity, which contributes to the evolutionary divergence between different miRNA genes, including homologous miRNAs (especially for multicopy pre-miRNAs). Indeed, although varied loop sequences exist, we still found the potential nucleotide characteristics in the 5′ and 3′ ends of the loops (Figure 4, Figure 5, Figure S2 in File S1 and Table S2 in File S1). Dominant nucleotides, such as uracil and guanine, are present at higher frequencies at the terminal ends, with these biased nucleotide compositions possibly influencing Dicer cleavage and contributing to the phenomenon of multiple isomiRs. While many isomiRs can be produced from a miRNA loci, only several isomiRs are dominantly expressed [21], [24], [33].

Some let-7 sequences are located in gene clusters with homologous miRNAs or other miRNAs, such as with the mir-125 and mir-99 gene families. Loops of clustered miRNA gene families may have different lengths, suggesting the length difference of loops from different miRNA genes (Table S3 in File S1). Significant differences in 5′ and 3′terminal nucleotide compositions are noted among these clustered miRNAs, with both uracil and guanine dominating in the two terminus ends (Table S2 in File S1). Compared to the stable length distributions of miRNAs, rapid loop sequence evolution can drive miRNA gene evolution and may further affect isomiR expression profiles during pre-miRNA processing [11], [15]. Expression analysis indicates stable isomiR expression profiles [21], [25], even across different tissues and animal species, suggesting stable Drosha and Dicer cleavage during miRNA processing and maturation. Furthermore, loop sequences also show higher levels of nucleotide divergence between homologous miRNA genes, especially between multicopy pre-miRNAs (Figure 2 and Figure S1 in File S1). These findings suggest that nucleotide divergence does not influence cleavage precision, but that nucleotide bias in the 5′ and 3′ ends of the loop potentially influences miRNA maturation.

Herein, canonical miRNA loop sequences were collected, with variations in the loops potentially based on the phenomenon of multiple isomiRs, with the canonical miRNA sequences not necessarily the most dominant sequence. However, according to length distributions and terminal end nucleotide compositions, it can be concluded that loop sequences tend to be longer in higher animal species, with rapid evolution of the loop sequences further driving miRNA gene evolution. Stable isomiR expression profiles indicate relative cleavage, which may be closely related to the dominant nucleotide distributions. This study further enriches the understanding of miRNA biogenesis as it relates to loop sequences across different animal species and among homologous miRNAs, particularly considering the phenomenon of multiple isomiRs.

## Supporting Information

File S1 **Figure S1. Examples of neighbor-joining tree of the loop and pre-miRNA sequences in the let-7 gene family. Figure S2. Sequence logo of loop sequences and miRNA sequences.** (A) Sequence logo of loop sequences from single let-7 genes and (B) sequence logo of relevant mature miRNAs (including miR-#-5p and miR-#-3p). **Table S1. Loop sequence length distribution from** **Figure 2B** **. Table S2. Nucleotide composition and frequency. Table S3. 95% confidence interval (CI) and difference between length distributions of let-7 and related clustered miRNAs. Table S4. Effect on minimum free energy (MFE) when changing loop sequence lengths in hsa-let-7a-1.**
(DOC)
