# Supplementary material for: Genetic Analysis of Loop Sequences in the Let-7 Gene Family Reveal a Relationship between Loop Evolution and Multiple IsomiRs
Source: PLoS One. 2014 Nov 14;9(11):e113042. doi: 10.1371/journal.pone.0113042 (PMC4232593; doi:10.1371/journal.pone.0113042)
Supplement: File S1 — Figure S1. Examples of neighbor-joining tree of the loop and pre-miRNA sequences in the let-7 gene family. Figure S2. Sequence logo of loop sequences and miRNA sequences. (A) Sequence logo of loop sequences from single let-7 genes and (B) sequence logo of relevant mature miRNAs (including miR-#-5p and miR-#-3p). Table S1. Loop sequence length distribution from Figure 2B . Table S2. Nucleotide composition and frequency. Table S3. 95% confidence interval (CI) and difference between length distributions of let-7 and related clustered miRNAs. Table S4. Effect on minimum free energy (MFE) when changing loop sequence lengths in hsa-let-7a-1. (DOC) [file pone.0113042.s001.doc]

**Figure S1. Examples of neighbor-joining tree of the loop and pre-miRNA sequences in the let-7 gene family.**

**Figure S2. Sequence logo of loop sequences and miRNA sequences.**

(A) sequence logo of loop sequences from single let-7 genes; (B) sequence logo of relevant mature miRNAs (including miR-#-5p and miR-#-3p).

**Table S1. The difference of length distribution of loop sequences in Figure 2B.**

| **Groups** | **Difference** | ***P*_adj_** | **Groups** | **Difference** | ***P*_adj_** |
| --- | --- | --- | --- | --- | --- |
| 1/2 | -13.73 | 0.0000 | 2/3 | 7.45 | 0.0000 |
| 1/3 | -6.28 | 0.0001 | 2/4 | 8.98 | 0.0000 |
| 1/4 | -4.75 | 0.0308 | 3/4 | 1.53 | 0.7852 |

“1” indicates let-7 in human, “2” indicates let-7 in Drosophila, “3” indicates let-7 in Urochordata, Echinodermata and Hemichordata, and “4” indicates let-7 in Lophotrochozoa.

**Table S2. Nucleotide composition and frequency.**

| **miRNA (± nt or +/- nt)** | **A (%)** | **U (%)** | **C (%)** | **G (%)** | ***χ^2^*, *P*** |
| --- | --- | --- | --- | --- | --- |
| clustered let-7 family (±5) | 647(34.23%) | 491(25.98%) | 110(5.82%) | 642(33.97%) | 301.41, 0.0001^*^ |
| clustered let-7 family (+3/-3) | 139(24.51%)/239(42.15%) | 229(40.39%)/194(34.22%) | 29(5.11%)/45(7.94%) | 170(29.98%)/89(15.70%) | 58.14, 0.0001 |
| clustered let-7 family (+1/-1) | 26(13.76%)/87(46.03%) | 116(61.38%)/84(44.44%) | 3(1.59%)/12(6.35%) | 44(23.28%)/6(3.17%) | 72.33, 0.0001 |
| clustered mir-99 family (±5) | 120(17.14%) | 190(27.14%) | 188(26.86%) | 202(28.86%) |  |
| clustered mir-99 family (+3/-3) | 7(3.33%)/52(24.76%) | 64(30.48%)/41(19.52%) | 34(16.19%)/64(30.48%) | 105(50.00%)/53(25.24%) | 65.66, 0.0001 |
| clustered mir-99 family (+1/-1) | 1(1.43%)/30(42.86%) | 9(12.86%)/15(21.43%) | 22(31.43%)/16(22.86%) | 38(54.29%)/9(12.86%) | 47.47, 0.0001 |
| clustered mir-125 family (±5) | 71(16.90%) | 161(38.33%) | 82(19.52%) | 106(25.24%) |  |
| clustered mir-125 family (+3/-3) | 15(11.90%)/17(13.49%) | 49(38.89%)/57(45.24%) | 28(22.22%)/19(15.08%) | 34(26.98%)/33(26.19%) | 2.47, 0.481 |
| clustered mir-125 family (+1/-1) | 3(7.14%)/4(9.52%) | 4(9.52%)/19(45.24%) | 25(59.52%)/14(33.33%) | 10(23.81%)/5(11.90%) | 14.69, 0.002 |
| let-7 family (±5) | 995(29.09%) | 841(24.59%) | 182(5.32%) | 1402(40.99%) |  |
| let-7 family (+3/-3) | 226(22.03%)/272(26.51%) | 393(38.30%)/378(36.84%) | 55(5.36%)/57(5.56%) | 352(34.31%)/319(31.09%) | 6.20, 0.102 |
| let-7 family (+1/-1) | 30(8.77%)/23(6.73%) | 209(61.11%)/279(81.58%) | 4(1.17%)/16(4.68%) | 99(28.95%)/24(7.02%) | 63.90, 0.0001 |

^*^ indicates the difference between clustered let-7 (±5), mir-99 (±5) and mir-125 (±5) gene families, others results in column of (***χ^2^*, *P***) are difference between +3 and -3 or +1 and -1. Moreover, among the three clustered gene families (+1 and -1), the relevant statistical results are: ***χ^2^* =** 147.18, *P* < 0.0001 and ***χ^2^* =** 51.26, *P* < 0.0001, respectively.

**Table S3. 95% confidence interval (CI) and difference between length distributions of let-7 and related clustered miRNAs.**

|  | **n** | **95% CI (nts)** | ***t*** | ***P*** |
| --- | --- | --- | --- | --- |
| let-7 miRNAs | 184 | 25.28-27.00 | 16.14 | 0.0000 |
| Clustered miRNAs | 125 | 14.50-16.40 |
| hsa-let-7 miRNAs | 10 | 25.04-31.76 | 5.77 | 0.0001 |
| hsa-clustered miRNAs | 5 | 12.54-18.26 |

let-7 miRNAs: all the let-7 sequences that have been identified with clustered miRNAs across different animal species; clustered miRNAs: miRNAs have close physical distances with let-7; hsa-let-7 miRNAs: all the human let-7 sequences that have been identified with clustered miRNAs; hsa-clustered miRNAs, miRNAs have close physical distances with let-7 in human.

**Table S4. Minimum free energy (MFE) through changing length of loop sequences in hsa-let-7a-1.**

| **Deleted Nucleotides** | **MFE** | **Deleted Nucleotides** | **MFE** | **Deleted Nucleotides** | **MFE** |
| --- | --- | --- | --- | --- | --- |
| 0 | -34.20 | 4 | -32.10 | 16 | -28.70 |
| 1 | -32.70 | 5 | -31.30 | 20 | -29.30 |
| 2 | -32.40 | 8 | -30.10 | 24 | -28.50 |
| 3 | -29.20 | 12 | -28.40 | 29 | -24.10 |

Deleted nucleotides are randomly performed in the loop sequences. The final loop sequence of hsa-let-7a-1 is 29 nts.
